# New Strategies for Innovative and Enhanced Meat and Meat Products

**DOI:** 10.3390/foods11050772

**Published:** 2022-03-07

**Authors:** Gonzalo Delgado-Pando, Tatiana Pintado

**Affiliations:** Institute of Food Science, Technology and Nutrition (CSIC), José Antonio Novais 10, 28040 Madrid, Spain; tatianap@ictan.csic.es

New strategies in the field of meat and meat product development are certainly needed in order to overcome not only the health-related problems these products might contribute to, but also from the perspectives of sustainability and the economy. 

Sustainability is now on the agenda of the United Nations member states, who in 2015 declared the sustainable development goals for 2030. The meat industry should be one of the food industries more concerned about this issue, as the low efficiency of animal production as well as environmental issues make this sector key in advancing towards food sustainability. In this Special Issue, Pintado and Delgado-Pando [1] reviewed the use of meat extenders as a way of contributing towards more sustainable meat products. The use of pulses, cereals, tubers, fruits, mushrooms, food by-products, and insects were evaluated as meat replacers or extenders in several types of meat products. Even though there are many of these ingredients that have been successfully employed for substituting meat content, there is need for further research where not only the product quality but also the consumer acceptance is jointly evaluated. A very interesting manuscript presented by Bakhsh et al. [2] proposed the use of plant-based meat analogues for tackling this sustainability issue. The authors use methylcellulose in different concentrations for the development of beef patty analogues of soy protein isolate and soy-based textured vegetable protein. These analogues were compared to a beef patty control in terms of physicochemical and sensory properties. Promising results were obtained in the patty with soy-based textured vegetable protein and 3% of methylcellulose, although a much lower hardness than the control patty was still an issue to improve. The use of by-products as ingredients in meat products can have a double purpose of increasing sustainability and improving the healthiness. Coffee silver skin (CSS) is a unique by-product of coffee roasting that is usually discarded, contributing to food waste. CSS was used in chicken burgers, contributing to the use of these by-products and improving the nutritional properties of the burgers: more fibre, minerals, and bioactive molecules [3]. Another proposal of using a food by-product was made by Summo et al. [4] that utilised oat hull, a by-product of oat milling, as a fat replacer in low-fat beef burgers. The authors found that a 100% substitution of fat from animal origin by this by-product generated burgers more appealing to the consumers with a higher juiciness and a softer texture. 

Meat and meat product consumption has been related to an increased risk of developing certain cancers and cardiovascular disease [5,6]. Saturated fat, naturally present in foods from animal origin, have been targeted as one of the issues towards CVD. Pintado and Cofrades [7] proposed a novel approach of substituting the pork backfat of dried fermented sausage with a mixture of oils from plant origin: olive and chia. The authors manufactured a beeswax oleogel and an emulsion gel as carriers of these oils and found an improved lipid profile and a good oxidative and microbiological status, irrespective of the carrier used. Other components of meat products related to health issues are the additives. An extensive review of clean label alternatives proposed by Delgado-Pando et al. [8] explored the idea of replacing the traditional additives with clean label alternatives. Even though the terminology is not yet properly defined, the ideas of consumers and industry were discussed. The origin of the additive, i.e., natural vs. synthetic (e.g., nitrites from green vegetables vs. synthetic nitrites), could be perceived as a good trait but the health problems associated with some additives do not distinguish if the substance is extracted from nature or synthesised in a laboratory, the chemical component remains the same. Nitrites have been related to the formation of N-nitroso compounds, known as human carcinogenic [8]. Tomovic et al. [9] explored the idea of using the *Juniperus communis* L. essential oil as an alternative for sodium nitrite in dry fermented sausages. The authors found that this essential oil could partially replace the use of nitrite as it provides significant antioxidant activity, maintaining the shelf life. Another additive that is being scrutinised is phosphate. Phosphates are widely used texturisers in meat products and have been related to increased CVD in people with chronic kidney disease. Although not harmful for healthy people, EFSA found that the exposure was higher than the acceptable daily intake for some population groups [8]. Goemaere et al. [10] studied the use of seven protein-based ingredients as phosphate replacers in emulsified meat products. The authors found that blood plasma and soy were superior in phosphate-free cooked sausages compared with sausages containing phosphates, in terms of texture, cooking yield, and stability. However, the authors admit that the meat matrix is important when selecting one ingredient or another as phosphate replacer. On the other hand, restructured ostrich ham was successfully formulated with a partial substitution of phosphates by iota carrageenan [11]. Another clean label alternative was proposed by Mancini et al. [12] who utilised common spices such as salt and garlic powder in rabbit burgers. They observed that these two ingredients played an important role in colour changes during storage and that higher garlic levels should be explored if a bacteriostatic effect is also intended. 

This Special Issue was completed with two research articles that will contribute to economic improvement by innovation and consumer information. From the latter, Yang et al. [13] did a thorough study of how providing nutritional information can boost the purchase intention of meat products by consumers in Taiwanese wet markets. This shows that sales could be improved by studying the information the consumer obtains during shopping. In terms of innovation, Hrbek et al. [14] proposed a technique for the authentication of meat and meat products, as well as meat adulteration, by using triacylglycerol profiling and DNA analysis. The authors proposed a direct analysis in real time, coupled with high-resolution mass spectrometry and combined with a multiplex polymerase chain reaction.
